# Cationization of *Eucalyptus* Kraft LignoBoost Lignin: Preparation, Properties, and Potential Applications

**DOI:** 10.1021/acs.iecr.1c04899

**Published:** 2022-03-07

**Authors:** Patrícia I. F. Pinto, Sandra Magina, Enkhjargal Budjav, Paula C. R. Pinto, Falk Liebner, Dmitry Evtuguin

**Affiliations:** † RAIZ—Forest and Paper Research Institute, Quinta de S. Francisco, Apartado 15, Eixo, 3801-501 Aveiro, Portugal; ‡ CICECO—Aveiro Institute of Materials and Department of Chemistry, University of Aveiro, Campus Universitário de Santiago, 3810-193 Aveiro, Portugal; § Department of Chemistry, Institute of Chemistry of Renewable Resources, University of Natural Resources and Life Sciences, Vienna (BOKU), Konrad Lorenz Straße 24, A-3430 Tulln, Austria

## Abstract

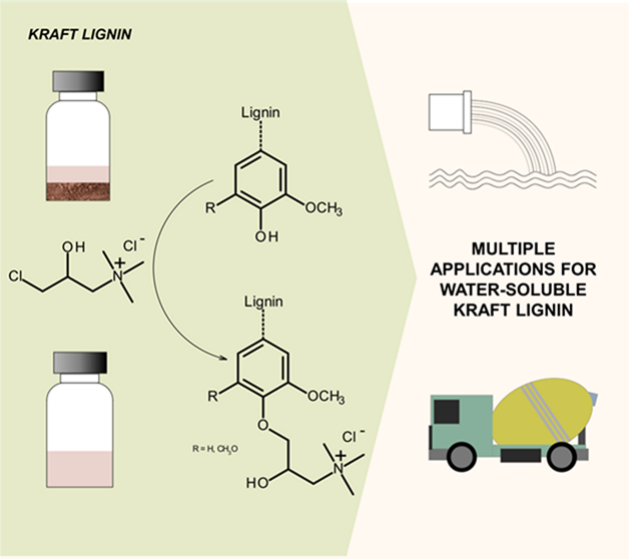

Current changes toward a more biobased economy have recently created tremendous renewed interest in using lignin as a valuable source for chemicals and materials. Here, we present a facile cationization approach aiming to impart kraft lignin water-solubility, with similar good features as lignosulfonates. *Eucalyptus globulus* kraft lignin obtained from a paper mill black liquor by applying the LignoBoost process was used as the substrate. Its reaction with 3-chloro-2-hydroxypropyl-trimethylammonium chloride (CHPTAC) in an aqueous alkaline medium was studied to assess the impact of different reaction conditions (temperature, time, educt concentration, molar CHPTAC-to-lignin ratio) on the degree of cationization. It has been shown that at pH 13, 10 wt % lignin content, 70 °C, and 3 h reaction time, a CHPTAC-to-lignin minimum molar ratio of 1.3 is required to obtain fully water-soluble products. Elemental analysis (4.2% N), size-exclusion chromatography (*M*_w_ 2180 Da), and quantitative ^13^C NMR spectroscopy of the product obtained at this limit reactant concentration suggest introduction of 1.2 quaternary ammonium groups per C9 unit and substitution of 75% of the initially available phenolic OH groups. The possible contribution of benzylic hydroxyls to the introduction of quaternary ammonium moieties through a quinone methide mechanism has been proposed. Since both molecular characteristics and degree of substitution, and hence solubility or count of surface charge, of colloidal particles can be adjusted within a wide range, cationic kraft lignins are promising materials for a wide range of applications, as exemplarily demonstrated for flocculation of anionic dyes.

## Introduction

Lignin is the second most abundant biopolymer on earth with an estimated annual growth of approximately 40 billion tons.^1^ Associated with the polysaccharides cellulose and hemicellulose in the cellular architecture of higher plants, lignin imparts strength, hydrophobicity, and resistance toward light-induced chemical and microbial degradation to our terrestrial vegetation, including wood.

Chemical pulping of wood is a global business satisfying the demand of our society for pulp, paper, and related products. Respective technologies are still mainly aiming to isolate fibrous cellulose, the primary component, from other wood constituents. The latter—among them lignin and hemicellulose—are solubilized into the aqueous reaction media to form “spent liquor”, the principal byproduct of chemical wood pulping. Nowadays, more than 90% of cellulosic pulp is produced by sulfate (or kraft) pulping using sodium hydroxide and sodium sulfide as pulping reagents. Less than 10% is produced by sulfite technologies using SO_2_ and inorganic bases as pulping reagents. Respective spent liquors contain water-soluble lignins (lignosulfonates), which find use in multiple applications.

Spent liquor (or black liquor) of kraft pulping is dark-colored and malodorous. It is typically upconcentrated, partly blended with auxiliaries, and subjected to burning to recover process chemicals, and energy. However, being aware of the relatively low heating value of black liquors,^2^ economic benefits from lowering the lignin load to the recovery boilers but also boosted by current efforts toward a more biobased economy, paper mills are currently exploring new opportunities for enhancing commercialization of byproducts, in particular of lignins.

The idea itself is not new since lignosulfonates, the major byproducts of sulfite pulping, have been marketed since decades for a variety of large-scale applications. This includes the areas of emulsifiers (asphalt, inks, and waxes), dispersants (drilling fluids, clay, ceramics, dyes, and pigments), binders (pelletizing of animal feed, ceramics, dust control, confectioning of fertilizers), and concrete plasticizing agents or flooring adhesives. Most of these applications, however, rely on good water-solubility of the respective lignins. Therefore, this business was hitherto largely restricted to lignosulfonates.

Kraft lignins, the byproducts of kraft pulping, are water-soluble at high pH only and precipitate upon dilution or lowering the pH. Therefore, they are not suitable for many applications. On the other hand, kraft pulping has nowadays clearly outpaced the various sulfite pulping technologies. Efficient measures are therefore needed that are capable of imparting good water-solubility across the entire pH scale to lignins, rendering them competitive with lignosulfonates. This could bridge gaps that may arise in the future from receding sulfite pulping business but could pave the way for novel mass or niche applications.

Measures capable of adding electrically charged moieties to lignin would probably be the most straightforward approach for increasing its polarity. Among the previously tested strategies targeting the introduction of negatively charged moieties, sulfonation is one of the more facile and understandably most promising approaches to obtain products competitive with lignosulfonates.^3−7^ However, the existence of negative charges in materials carrying phenolate, carboxylate, or sulfonate moieties is typically limited to a certain (alkaline) pH range. In acidic media, these moieties exist mainly in the nondissociated form, with their extent depending on the respective p*K*_S_ values. The introduction of pH-independent, positively charged moieties (cationization) is therefore particularly appealing. This is even more the case since a variety of applications could additionally benefit from physical interactions with negatively charged surfaces, which are abundant in nature. It includes charge-stabilization of inorganic emulsions, modulation of electrical double-layer properties, or interactions with microorganisms, viruses, or living tissues.^8,9^

The inability of most nonmetal main group elements to form stable cations at economically feasible conditions narrows down the choice drastically to quaternary ammonium groups, i.e., nitrogen atoms carrying four organic residues. Grafting of such moieties is typically accomplished by derivatization reagents composed of a reactive group, a short-chain spacer, and the quaternary ammonium group. Commonly used reagents are 3-chloro-2-hydroxypropyl-trimethylammonium chloride (CHPTAC), (2,3-epoxypropyl) trimethylammonium chloride (EPTAC), diallyldimethylammonium chloride (DADMAC), [2-(methacryloyloxy)ethyl]trimethylammonium chloride (METAC), and (2-hydrazinyl-2-oxo-ethyl)-trimethylazanium chloride (GT).^10,11^

The cationization of inorganic and organic substrates including biopolymers using reagents such as those mentioned above is common practice. In papermaking for example, it is extensively used to improve the retention of starch, in particular, at the wet end of the process. It is achieved by increasing its physical bonding capabilities toward anionic surfaces abundant on both the fiber raw materials and fillers.^12^ Cationic cellulose nanofibrils were recently used to produce highly porous (37–48%) yet surprisingly strong cellulose nanopaper (*E* = 10 GPa, σ_max_ = 200 MPa), which featured high water absorbency (750 g g^–1^) and surface charge density.^13^ In another study, cationic nanofibrillated cellulose (cat-NFC) was shown to feature strong antimicrobial activity against the human pathogens *Micrococcus luteus*, *Escherichia coli*, and *Candida oleophila*. The respective material was prepared by (i) cellulose treatment with EPTAC, (ii) nanofibrillation, and (iii) redox-initiated graft polymerization of METAC on the surface of cat-NFC in the aqueous dispersion state.^14^

The potential of biopolymer cationization can also be demonstrated in the example of chitosan, a partially deacetylated derivative of chitin. While chitosan is insoluble in water at pH >6.4, introduction of quaternary ammonium groups extends its water-solubility to the full alkaline range.^15,16^ Cationization also increases its antimicrobial activity^17,18^ and improves its properties as a drug carrier upon crossing the epithelia.^19,20^ Cationization of lignin has been hitherto almost exclusively considered for wastewater treatment, requiring relatively small quantities of flocculation agents^21−32^ or coagulation aids.^27,33,34^ Respective studies have covered a broad range of pollutants including sulfate^27^ or nitrate ions,^22^ heavy metal complexes like chromates,^28^ clay particles like kaolin^23,27^ or bentonite,^29,30^ organic dyes,^21,24−26,32,34^ or humic acids.^27,35^ Softwood kraft lignin^21,26,29−32^ and soda lignin^22−25,35^ have been the major sources of materials for these investigations, while organosolv lignin^27,28^ or enzymatic hydrolysis lignin^27^ was used in only a few studies. Beyond flocculants, only sparse literature exists, showing that lignosulfonates modified by the introduction of quaternary ammonium groups can improve the dispersion capacity of polycarboxylate ethers in clay-containing cement paste^36^ or suggesting that softwood kraft lignin polymerized with METAC as the paper strength additive.^37^ Complementing a comprehensive study investigating the impact of various reaction parameters on the properties of pine kraft lignin modified by EPTAC,^26^ the suitability of various lignin preactivation and or formulation approaches has also been tested. This includes attempts to increase either the number of reactive sites for introduction of quaternary ammonium groups, such as by preceding phenolation,^22^ or the charge density on the surface of micron-sized particles prepared by a Mannich-type reaction of organosolv lignin with formaldehyde and Girard T’s reagent.^28^

To the best of our knowledge, this study investigates for the first time the conversion of hardwood kraft lignin into cationic products highly soluble in water over a wide pH range. Aiming to exclude the interference of byproducts and working with a well-defined lignin separated and purified by cutting-edge technologies, black liquor from a kraft mill was subjected to a pilot-scale LignoBoost process. Besides the impact of different reaction conditions on the degree of substitution (DS) and solubility, selected products were subjected to instrumental analysis, such as size-exclusion chromatography (SEC), nuclear magnetic resonance spectroscopy (^1^H, ^13^C), X-ray photoelectron spectroscopy (XPS), Fourier transform infrared (FT-IR) spectroscopy, and thermogravimetric analysis (TGA, derivative thermogravimetry (DTG)). Furthermore, hygroscopicity, antioxidant activity, and flocculation activity were assessed in preliminary experiments.

## Experimental Section

### Materials

*Eucalyptus globulus* kraft lignin was kindly provided by The Navigator company (Aveiro, Portugal). It was isolated from black liquor of a pulp mill using the LignoBoost pilot plant facilities of RISE (Stockholm, Sweden). Prior to cationization, the source material was vacuum-dried (100 mbar, 30 °C) for 7 days.

Acetone-*d*_6_ (99.5 atom % D), deuterium oxide (99.8 atom% D), iron(II) chloride hexahydrate (≥99%, FeCl_3_·6H_2_O), water (high-performance liquid chromatography (HPLC) grade), sodium nitrate (99%, NaNO_3_), phosphorus pentoxide (≥99%, P_4_O_10_), and 2,4,6-tris(2-pyridyl)-*s*-triazine (TPTZ, 99%) were purchased from Acros Organics (Geel, Belgium). Sodium polyethylene sulfonate (Pes-Na, 0.001 N) was provided by BTG Instruments AB (Säffle, Sweden). l-Ascorbic acid, 1,4-dioxane, sodium chloride, sodium hydroxide, and sulfuric acid were all of ACS grade and supplied by Fisher Chemical (Loughborough, U.K.). Hydrochloric acid and sodium acetate trihydrate were of ACS grade and obtained from Panreac (Darmstadt, Germany). Pullulan standards covering the molecular weight range of 738–48 000 Da were from Polymer Laboratories (Church Stretton, U.K.), and 3-chloro-2-hydroxypropyl-trimethylammonium chloride (CHPTAC, 65 wt % in H_2_O) was purchased from TCI Europe N.V. (Zwijndrecht, Belgium). Dialysis tubing (benzoylated cellulose, cutoff 2000 Da) and Remazol Brilliant Blue (reactive dye 19) were obtained from Sigma-Aldrich (Lisbon, Portugal). All chemicals were used as received unless mentioned otherwise. All treatments with water refer to deionized qualities (EC ≤ 2.67 μS m^–1^).

### Preparation of Cationic Kraft Lignin

A solution of 5.0 (23.6 mmol) or 10.0 g (47.3 mmol) of *E. globulus* kraft lignin (*M*_r_ = 211.4 g mol^–1 ^^38^) in 50 mL of 1 M sodium hydroxide (50 mmol) was placed in a nitrogen-flushed three-necked round-bottom flask equipped with a gas inlet, a condenser, and rubber sealing. Defined volumes (2.5–22.5 mL) of a 65 wt % aqueous solution (ρ_20 °C_ = 1.17 mg mL^–1^) of CHPTAC equivalent to CHPTAC-to-lignin (C/L) molar ratios of 0.4–3.8 were slowly added within a time period of 15 min. The reaction mixture was kept at room temperature (RT) (QL-8, QL-10) or heated to 70 °C under continuous stirring for 1–24 h. After cooling, the aqueous alkaline solution was neutralized (ca. pH 7) using semidilute sulfuric acid and transferred into a dialysis membrane (cut-off 2000 Da). Two-step dialysis was performed using dilute brine (2 and 1 g L^–1^). The obtained solution of purified cationic lignin was then freeze-dried using a Lyovapor L-200 (Büchi, Flawil, Switzerland) and kept in a desiccator over phosphorous pentoxide until further use. Detailed reaction conditions and labeling of the quaternized lignins (QL) can be found in Table 1.

**Table 1:** Impact of Various Reaction Parameters on the Attainable Degree of Substitution as Determined by Nitrogen Elemental Analysis

	molar reactant ratio	concentration					
sample code	CHPTAC/lignin (C/L ratio)	lignin (wt %)	NaOH (mol L^–1^)	reaction time (h)	temperature (°C)	nitrogen content (wt %)	degree of substitution (mol mol^–1^)
QL-1	0.4	10	1	3	70	1.6	0.3
QL-2	0.6	10	1	3	70	3.2	0.7
QL-3	1.3	10	1	1	70	3.5	0.9
QL-4	1.3	10	1	2	70	4.0	1.1
QL-5	1.3	10	1	3	70	4.2	1.2
QL-6	2.6	10	2	1	70	4.5	1.3
QL-7	2.6	10	2	3	70	4.4	1.3
QL-8	2.6	10	2	3	25	4.1	1.1
QL-9	2.6	10	2	4	70	4.5	1.3
QL-10	2.6	10	2	24	25	4.5	1.3
QL-11	2.6	20	2	3	70	3.8	1.0
QL-12	3.8	10	3	3	70	5.1	1.7

### Characterization of the Parent and Modified Lignins

Ash contents of the parent and selected cationic lignins were determined gravimetrically. In brief, a defined amount of the oven-dried samples (ca. 1 g) was weighed into precalcined crucibles and placed in a muffle furnace (525 ± 25 °C) for 5 h. After cooling in an anhydrous atmosphere (desiccator, P_4_O_10_), the weights of the remaining materials were determined at a resolution of 0.1 mg and used to calculate the respective ash contents as described elsewhere.^39^

Elemental analyses were conducted using an Elementar vario MAX cube elemental analyzer (Elementar Analysensysteme GmbH, Langenselbold, Germany).

The pH-dependent water-solubility of the cationized lignins at 30 °C was exemplarily studied for the sample QL-5. A series of concentrations (20, 100, 500, and 1000 g L^–1^) was prepared using deionized water (pH 7). Adjustment of pH to the upper and lower limit values of 12 and 2 was accomplished using 1 M NaOH or 0.4 M H_2_SO_4_. After 1 h of equilibration at the respective pH (30 °C, continued stirring), the solutions were filtered using dried, preweighed filters. Based on the mass of insolubles remaining in the oven-dried (105 °C, 12 h) filter, the weight fraction of cationic lignin soluble at the respective pH of interest was determined and used to calculate the solubility.

**Figure 1:**
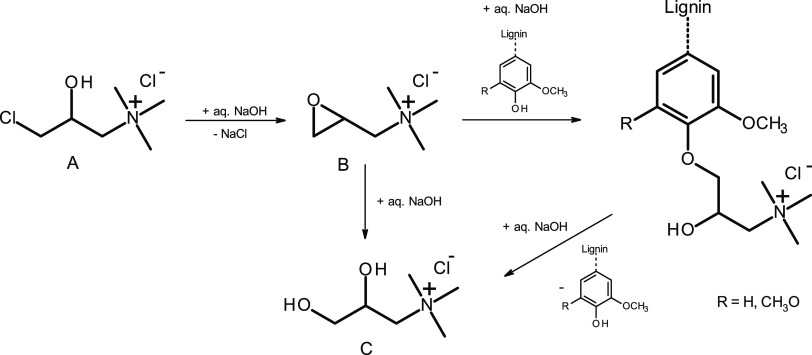
Lignin derivatization with CHPTAC (A) proceeding via the formation of EPTAC (B) in an aqueous alkaline medium to introduce quaternary ammonium moieties and to impart pH-independent water-solubility to lignin. The glycol derivative C is a potential major byproduct that can be formed in a competing reaction at stronger alkaline conditions.

Degree of substitution (DS), i.e., the count of quaternary ammonium moieties introduced per C9 “repeating unit” of lignin, was calculated from the nitrogen contents according to eq 1 as detailed previously.^40^ Here, N is the nitrogen content determined by elemental analysis (wt %), 211.4 is the molecular weight of the “C9 repeating unit” of the *E. globulus* kraft LignoBoost lignin used (g mol^–1^, cf. Results and Discussion section),^38^ 14 is the molecular weight of nitrogen (g mol^–1^), and 151.6 is the molecular weight of the newly introduced *N*,*N*,*N*-trimethyl-*N*-(2-hydroxypropyl)ammonium chloride moieties (C_6_H_14_NOCl, Figure 1). After elimination of equal units, the dimensionless value of the degree of substitution (DS) is obtained.
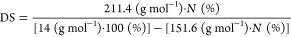
1Molecular weight characteristics were studied by size-exclusion chromatography (SEC) using a PL-GPC 110 system (Polymer Laboratories Ltd., Church Stretton, U.K.) configured with two PL aquagel-OH MIXED 8 μm 300 × 7.5 mm^2^ columns, a PL aquagel-OH Guard 8 μm precolumn, and an refractive index (RI) detector. Experimental conditions were an isocratic flow (0.9 mL min^–1^) of 0.1 M aqueous sodium nitrate at 36 °C. Samples were prepared by dissolving aliquots of selected cationic lignins in the mobile phase to obtain a concentration of about 8 mg mL^–1^. All solutions were filtrated through a 0.2 μm nylon filter prior to analysis. Calibration was performed using pullulan standards (Polymer Laboratories Ltd.) covering the molecular weight range of 738–48 000 Da.

FT-IR spectra were recorded using a TENSOR II FTIR spectrometer (Bruker Optik GmbH, Ettlingen, Germany) equipped with a horizontal attenuated total reflectance (ATR) cell. Freeze-dried samples stored in an anhydrous atmosphere (desiccator, P_4_O_10_) were used. Background-corrected spectra were generated in the absorbance mode, in the range of 400–4000 cm^–1^, at the resolution of 4 cm^–1^, and collecting 20 scans per sample.

^1^H NMR spectra were recorded on a Bruker Avance II 400 instrument (400.13 MHz) equipped with a 5 mm observed broadband probe head (BBFO) with *z*-gradients at room temperature using standard Bruker pulse programs. All samples were solubilized in D_2_O at concentrations of about 2 mg mL^–1^. Aiming to follow the conversion of CHPTAC under the conditions of lignin derivatization, three samples of CHPTAC in D_2_O were treated with NaOH (2 mg mL^–1^) at different temperatures (RT, 50, 70 °C) for different periods of time prior to NMR analysis.

Quantitative ^13^C NMR spectroscopy was carried out using a Bruker AVANCE III 300 spectrometer operating at 75.47 MHz (298 K), with 90° pulse, 12 s relaxation delay, and collecting 18 000 scans. The sample concentration in D_2_O was approximately 25% w/v (10 mm NMR tube), and acetone-*d*_6_ was used as an internal standard. For the sake of comparison, a quantitative ^13^C NMR spectrum of the parent *E. globulus* kraft lignin can be found elsewhere.^38^

X-ray photoelectron spectroscopy (XPS) of a representative cationized lignin sample (QL-5) was conducted at the Centre for Mechanical Technology and Automation (TEMA, University of Aveiro) using an ultrahigh-vacuum (UHV) instrument (SPECS GmbH, Berlin, Germany) equipped with a hemispherical electron energy analyzer (SPECS Phoibos 150), a delay-line detector, and a monochromatic X-ray source (Al Kα = 1486.74 eV). The system is operated at a base pressure of 2 × 10^–10^ mbar. The sample was placed on carbon tape before evacuation. High-resolution spectra were recorded at normal emission take-off angle and a pass energy of 20 eV, which provides an instrumental peak broadening of 0.5 eV. All binding energies were referenced to the first component of the C 1s core level (284.5 eV, C sp^2^).

Thermogravimetric analyses (TGA, DTG) of the parent and selected cationic lignins were carried out using a SETSYS Evolution instrument (Setaram Inc., Caluire, France) equipped with a DSC plate rod. A defined amount of the samples was placed into an alumina crucible and heated at 10 °C min^–1^ from 25 to 1000 °C in a nitrogen or oxygen atmosphere (200 mL min^–1^). A blank (empty crucible, reference) was treated in the same way for both types of gases (N_2_, O_2_). Temperature and heat flow calibrations were conducted using the melting points of four standards (In, Pb, Al, and Au) at three different heating rates (5, 10, and 15 °C min^–1^).

ζ potentials were measured using a Malvern zetasizer NANO-ZS ZEN 3600 (Malvern Instruments Ltd., Malvern, U.K.). Duplicate measurements of each sample (0.2 g L^–1^) were conducted using a folded capillary cell (Malvern Instruments Ltd., Malvern, U.K.). Evaluation was accomplished from the electrophoretic mobility data using the Smoluchowski model (Zetasizer 7.11 software). Results were expressed as average values.

Charge densities of selected cationic lignins were determined using a Mütek PCD-05 particle charge detector (BTG Instruments AB, Säffle, Sweden). Aliquots of 10 mL of respective lignin solutions (0.5 g L^–1^) were titrated against Pes-Na (0.001 N) solution. Duplicate measurements were performed, and average values were reported.

Moisture sorption analyses for selected cationic lignins were conducted at two levels of relative humidity (RH) and 25 °C. Freeze-dried samples were additionally dried over phosphorus pentoxide for 24 h prior to the experiments. The test conditions were established by placing saturated solutions of magnesium chloride hexahydrate (35% RH) and potassium chloride (78% RH) into desiccators and allowing them to equilibrate with the surrounding atmosphere for 12 h. After having exposed the samples to the respective test environment, their weight gain was determined after 30 min, 1 h, 5 h, 24 h, 4 days, 5 days, 8 days, 11 days, 19 days, and 27 days. Both RH and temperature were continuously recorded using an EL-USB-2 data logger (Lascar Electronics, Whiteparish, U.K.).

Antioxidant activities of both parent and selected cationic lignins were studied using the ferric reducing antioxidant power (FRAP) assay according to Benzie et al.^41^ This method reports reduction of Fe^3+^ to Fe^2+^ by polyphenols in an acidic medium through formation of a colorful complex with TPTZ that can be quantified photospectrometrically at 593 nm. In brief, 0.1 mL of the parent lignin or cationic lignin solutions (1 g L^–1^) dissolved in dioxane or water was placed into test tubes and incubated at 37 °C for 15 min. Then, 3.0 mL of the freshly prepared FRAP reagent was added. The absorbance of these mixtures was measured after 4 min reaction time against a blank containing 0.1 mL of dioxane or water using a Thermo Scientific Multiskan GO microplate spectrophotometer (Thermo Fisher Scientific, Vantaa, Finland). The FRAP reagent was prepared with 50 mL of acetate buffer (300 mmol L^–1^, pH 3.6), 5 mL of TPTZ (10 mmol L^–1^ in 40 mmol L^–1^ HCl), and 5 mL of FeCl_3_·6H_2_O (20 mmol L^–1^). The relative activities of the samples were calculated from the calibration curve of the standard l-ascorbic acid, which was established from a respective concentration series (50–1000 μmol L^–1^) treated with the FRAP reagent under the same experimental conditions. Mean values of triplicates were reported as the FRAP value, in μmol L^–1^.

Flocculation activity was assessed as described elsewhere,^26^ by studying the kinetics of the removal of an anionic model dye (Remazol Brilliant Blue R) from the solution state (100 mg L^–1^, pH 7) upon addition of selected cationic lignins. In brief, concentration series (50, 100, 200, 350, 400, 500, 600, and 800 mg L^–1^) of selected cationic lignins were prepared from respective stock solutions (2 g L^–1^, pH 7). The flocculation experiments (30 °C, water bath) were started by adding a defined volume of QL solution to 10 mL of the dye solution. After 30 min of continuous stirring at room temperature and 30 min of sedimentation, the mixture was centrifuged at 10 000 rpm for 10 min using a Thermo Scientific Fresco 21 microcentrifuge (Thermo Fisher Scientific, Osterode am Harz, Germany). The supernatant was subjected to photospectrometric analysis using a Thermo Scientific Multiskan GO microplate spectrophotometer (Thermo Fisher Scientific, Vantaa, Finland). Based on a calibration curve, the dye concentrations in the collected supernatants were calculated from their adsorption values using the absorption maximum of the model dye at λ = 595 nm. These values were then used to calculate the percentage of dye removal according to eq 2.
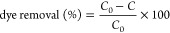
2where *C*_0_ and *C* are the concentrations (mg L^–1^) of dye solutions before and after adding the cationic lignins.^42^

## Results and Discussion

### Preparation and General Characteristics of Cationic Kraft Lignin

Industrial-scale chemical conversion of kraft lignins into valuable products featuring good water-solubility across the entire pH scale for large-scale applications requires an approach that meets both economic and ecological demands. Therefore, an aqueous process requiring mild reaction conditions and relatively low-cost modification reagents was envisaged.

3-Chloro-2-hydroxypropyl-trimethylammonium chloride (CHPTAC) is a compound that fits well to this concept. It is composed of a reactive site suitable for grafting onto lignin and a polar quaternary ammonium group. Both groups are linked to each other through a flexible short C3 alkyl chain carrying a polar hydroxyl group in the β-position, further facilitating the targeted water-solubility of the modified lignins. In the aqueous alkaline medium, CHPTAC forms the more reactive (2,3-epoxypropyl) trimethylammonium chloride (EPTAC), while free phenolic hydroxyl groups present in kraft lignins are converted to phenolate moieties. At room temperature, the latter can engage in S_N_2 epoxide ring-opening of EPTAC to afford lignin carrying the desired covalently attached 2-hydroxy-3-(*N*,*N*,*N*-trimethylammonium)-propyl moieties (Figure 1). In a competing reaction, the glycol derivative C can be formed as a major byproduct,^43^ which may reduce the content of the active modification reagent but should not remain in the product after dialysis. The onward reaction of compound C to oligo(ethylene glycol) derivatives is theoretically possible; however, this usually occurs only in water-deficient systems.^44^ It is worth mentioning that isolation of the modified lignin from the alkaline solution by evaporation, i.e., without neutralization, may reverse etherification under the formation of glycol C (Figure 1), as recently shown for cationization of hemicelluloses.^45^

*E. globulus* kraft lignin obtained from black liquor by applying the LignoBoost process was used as a substrate for all experiments of this study. As targeted by selection of this isolation and purification procedure, the parent lignin was low in ash content (1.42 wt %). According to elemental and functional group analyses,^38^ the source lignin had low nitrogen (0.20 wt %) and sugar (1.38 wt %) contents, while the mass fractions of phenolic (3.93 mmol g^–1^) and aliphatic hydroxyl groups (3.03 mmol g^–1^) as well as the ratio of syringyl-to-guaiacyl moieties (3.74) were in the typical range of hardwood lignins.^46^ Based on all analytical data, a C9 formula of C_9_H_6.76_O_2.95_S_0.18_(OCH_3_)_1.41_(OH_phen_)_0.83_(OH_aliph_)_0.64_ was calculated, equivalent to a C9 molecular mass of 211.4 g mol^–1^.

Variation of reaction parameters (for a comprehensive overview, see Table 1) to determine optimum conditions with regard to product solubility, yield, reaction time as well as consumption of chemicals revealed that a molar C/L ratio of ≥1.3 is required to afford products completely soluble in water. At ratios below that value, products were only partly soluble in water. In the example of sample QL-5, it was demonstrated that the excellent water-solubility of cationic hardwood lignins beyond C/L ratios of 1.3 is independent of the pH (test range pH 2–12), even up to a concentration of 450 g L^–1^.

**Figure 2:**
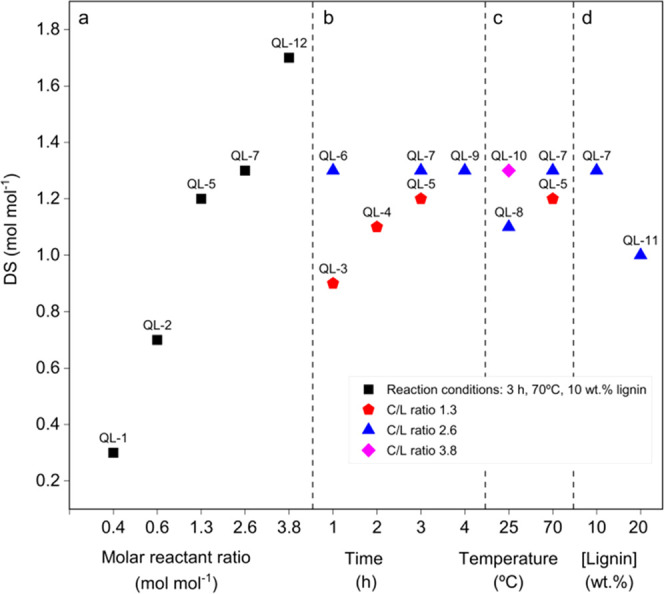
Effects of process parameter variation: (a) CHPTAC-to-lignin molar ratio (0.4–3.8 mol mol^–1^); (b) reaction time (1–4 h); (c) temperature (25, 70 °C); (d) lignin concentration (10, 20 wt %) on the degree of substitution of free lignin phenolic groups by quaternary ammonium moieties.

Nitrogen content analysis of the products confirmed that the degree of substitution (cf. formula 1) increases almost linearly for the lower range of tested C/L molar ratios (0.4, 0.6, and 1.3). At higher ratios, the increase in DS is less pronounced, as is evident when raising the C/L ratio from 1.3 to 2.6. The somewhat higher DS of 1.7 obtained at a CHPTAC-to-lignin ratio of 3.8 might be a result of the stronger alkaline conditions employed (3 M instead of 1 M aqueous NaOH, cf. Table 1; Figure 2a).

Extending the reaction time (70 °C) had a positive effect only on the CHPTAC-to-lignin ratio of 1.3. Doubling the time from 1 to 2 h resulted in a 0.5 wt % gain in nitrogen contents. Further prolonging the reaction time to 3 h had a smaller impact (0.2 wt % gain), and it seems that this effect levels off beyond that time. At a molar C/L ratio of 2.6, the highest attainable degree of substitution (DS 1.3) is reached already after 1 h of reaction time, and no further increase was observed when conducting the experiments for longer periods of time (Figure 2b).

Elevated temperature accelerates the introduction of quaternary ammonium moieties, as exemplarily demonstrated for a C/L ratio of 2.6. However, at room temperature, 24 h of reaction time is required to introduce 4.5 wt % nitrogen (DS 1.3), the same degree of substitution can be reached at 70 °C already after 4 h (Figure 2c).

The concentrations of the educts in the reaction mixture also seem to have an influence. This is evident from the significant drop of the nitrogen contents (4.6–3.8) when increasing the mass concentration of educts from 10 to 20 wt % (Figure 2d). Based on these findings, it is concluded that reasonable reaction conditions comprise the following: CHPTAC-to-lignin molar ratio of 1.3, 70 °C, 3 h of reaction time, 1 M NaOH, and 10 wt % lignin content.

Size-exclusion chromatography confirmed that both weight (*M*_w_) and number average (*M*_n_) molecular weight (relative to pullulan) of the products increased with the C/L ratio used and, hence, with the extent of cationization. While the parent lignin had an *M*_w_ of 1415 Da and *M*_n_ of 1295 Da,^38^ both values increased in the order QL-5 (C/L = 1.3, *M*_w_ 2180 Da, *M*_n_ 1760 Da) < QL-7 (C/L = 2.6, *M*_w_ 2350 Da, *M*_n_ 1890 Da) < QL-12 (C/L = 3.8, *M*_w_ 2380 Da, *M*_n_ 1920 Da).

This observed gain in weight average molecular mass corresponds approximately to an incorporation of 5.0 (QL-5) to 6.3 (QL-12) quaternary ammonium moieties (*M*_w_ 152.5 Da) per lignin molecule, which would be equivalent to a DS of 0.75–0.95. This range of substitution fits well the count of free phenolic groups of 0.83 per C9 unit in the parent lignin. It is evident that aliphatic hydroxyl groups are engaged in derivatization only at an excess of CHPTAC and at a significantly lower reaction rate. A comparison of the DS values calculated from the results of SEC with that derived from elemental (nitrogen) analysis (DS 1.2–1.7) suggests the presence of higher-molecular nitrogen-containing byproducts even after dialysis (cut-off 2000 Da). This is evident from the total hydroxyl group content, which is equivalent to 1.47 per C9 unit only (0.83 phenolic and 0.64 aliphatic OH groups). Ring-opening oligomerization of EPTAC under the stronger alkaline conditions of QL-12 synthesis could be one of the possible side reactions. Underestimation of DS by SEC is another likely reason for this discrepancy, which might be due to the relatively low molecular weight of the samples being close to the detection limit or the possible chemical interaction of the charged molecules with the stationary phase. Another plausible source of the imbalance observed between evaluation of DS by elemental analysis (DS 1.2–1.7) and that based on the number of reactive phenolic groups available (0.83 per C9) could be the formation of quaternary ammonium structures through a quinone methide mechanism.^47^ The latter could occur between benzyl-type lignin structures and the glycol derivative C of CHPTAC (Figure 1).

Irrespective of which DS values are considered, either calculated from the nitrogen contents or derived from SEC data, in both cases, they are significantly higher than the values previously reported by Md Noor et al.^23^ and Kong et al.^26^ for similar materials. Cationization of lignin from empty oil palm fruit bunches, for example, conducted at significantly higher molar C/L ratios of 5, 10, and 15 but otherwise similar conditions (24 h stirring at room temperature, 5 wt % lignin content in 0.2 M NaOH) afforded DS values of 0.20–0.30 only.^23^ Similarly, softwood kraft lignin purified according to the LignoForce technology and reacted with EPTAC at a molar reagent-to-lignin ratio of 2 (70 °C, 1 wt % lignin content, 1 h) afforded a DS of 0.24. However, higher DS values of 0.74 were reported for hardwood organosolv lignin modified in 10 wt % solution (60 °C, 20 h) using twice the stoichiometric amount of EPTAC.^27^ It is worth noting that the kraft lignin used in this study contained a certain proportion of tannins (e.g., ellagic acid),^38^ which might contribute to the observed DS due to the high abundance of phenolic groups.

**Figure 3:**
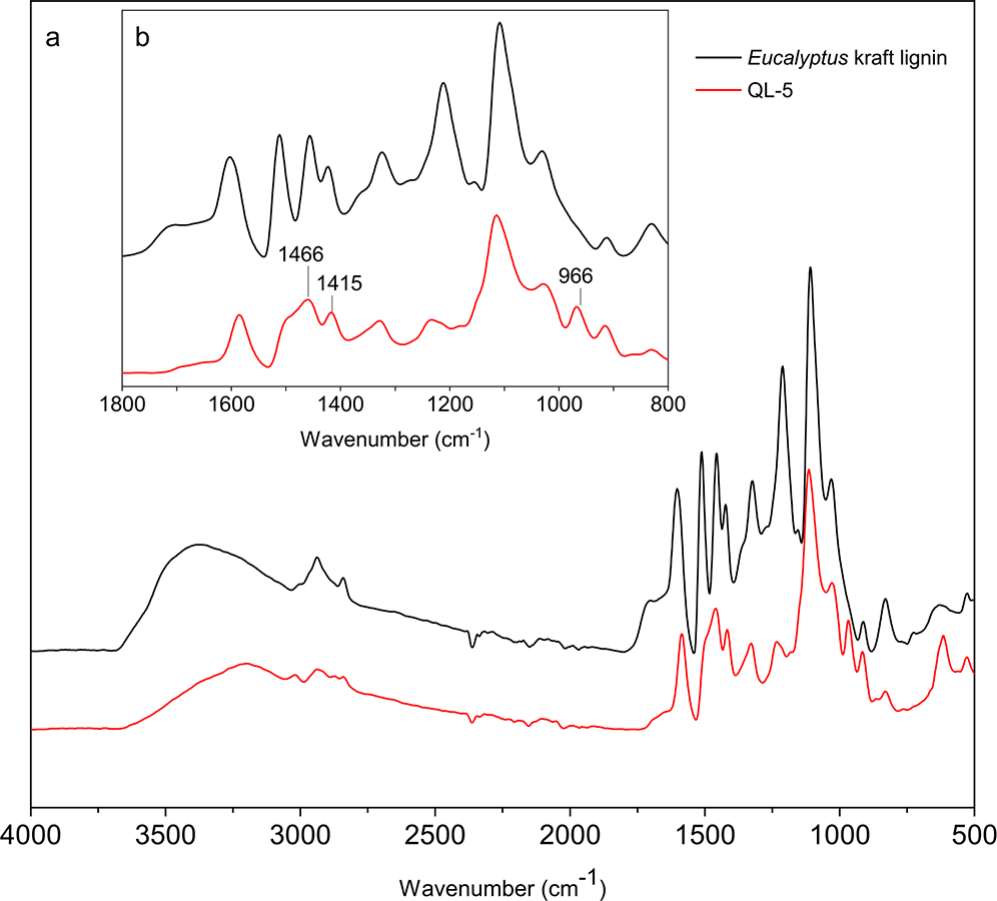
FT-IR spectra of *E. globulus* kraft lignin and its cationic derivative (sample QL-5). Full spectrum (a) and zoom of the wavenumber range of 800–1800 cm^–1^ (b).

The introduction of the quaternary ammonium moieties has been confirmed by FT-IR spectroscopy, as shown in Figure 3 (for the FT-IR spectra of QL-2 to QL-11, see Figure S1; cf. the Supporting Information). Different from the parent lignin, the modified lignin exemplarily studied (QL-5) shows a clearly reduced intensity of the broad band centered at 3420 cm^–1^. This is due to the reduced extent of O–H stretching vibrations in hydroxyl groups,^48^ as caused by the substitution reaction. The bands at 1466 and 966 cm^–1^ (methyl and methylene groups attached to the quaternary ammonium atoms) and 1415 cm^–1^ (C–N stretching vibrations), which are only present in the modified lignin, are further indicators of a successful modification.^23,26,35,49−51^ The wavenumbers of other peaks present in the spectrum of the cationic lignin are in agreement with the typical vibration band pattern of lignins and respective literature data: 2938 and 2840 cm^–1^ (stretching vibration of methyl and methylene groups^52^); 1603 cm^–1^ (aromatic skeletal vibration^49^); 1425 cm^–1^ (C–H in-plane deformation vibration superimposed by vibrations of the lignin aromatic skeleton^53^); 1328 cm^–1^ (C–H vibrations in syringyl-based structural units^54^); 1116 cm^–1^ (in-plane deformation vibration of C–H in syringyl moieties^49^); 1033 cm^–1^ (aromatic C–H in-plane deformation and C–O deformation in primary alcohols^49,55^); and 835 cm^–1^ (C–H out-of-plane deformation in positions 2 and 6 of syringyl groups^56^).

**Figure 4:**
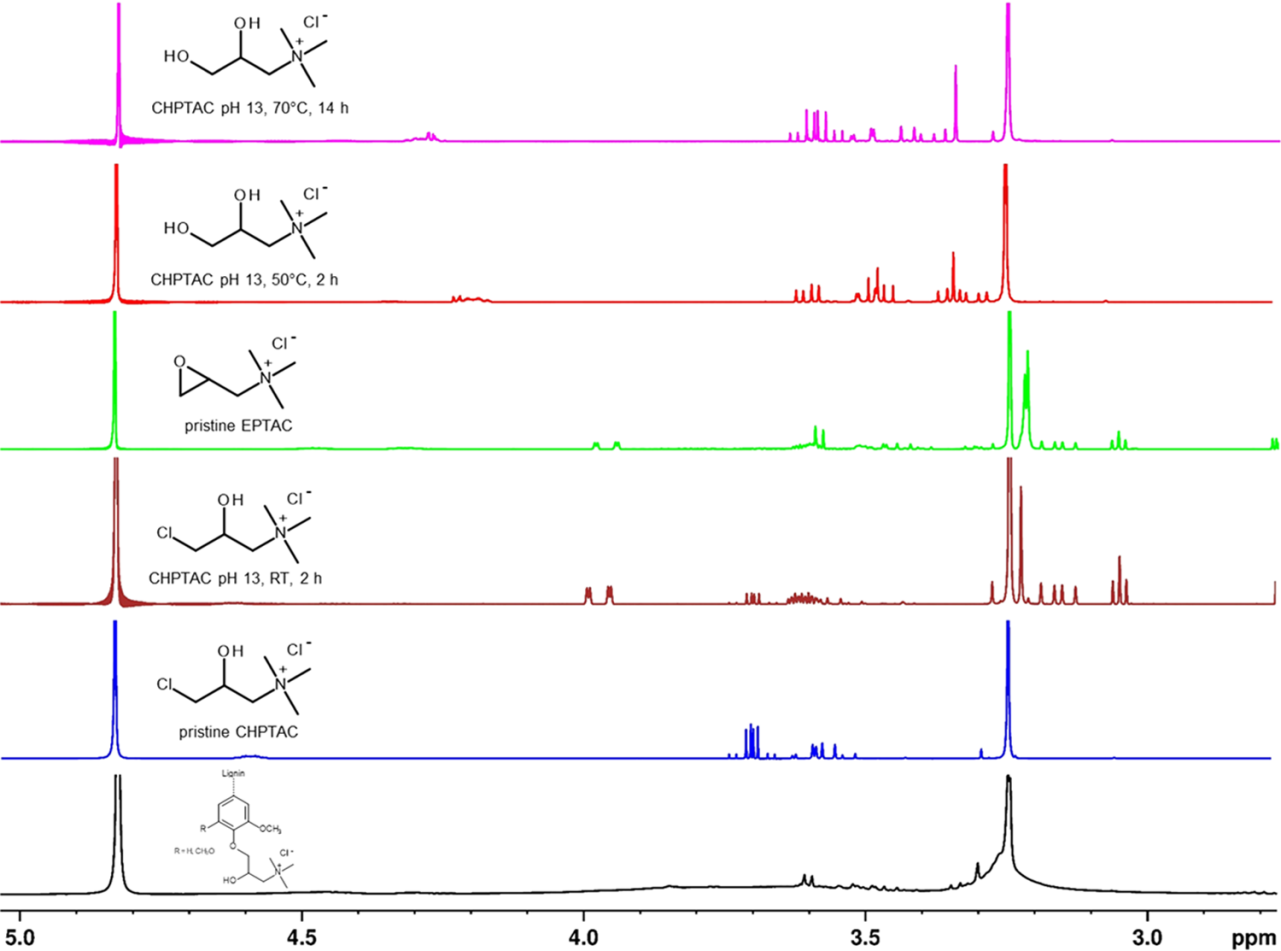
^1^H NMR spectra of cationized *E. globulus* kraft lignin (QL-5), CHPTAC, EPTAC, and three samples of CHPTAC in D_2_O at pH 12–13 and different temperatures (RT, 50, 70 °C) for different periods of time (2, 10 h) prior to NMR analysis.

^1^H NMR spectroscopy as exemplarily applied to sample QL-5 provided further evidence of the successful introduction of 2-hydroxy-3-(trimethylammonium) propyl moieties (Figure 4). Based on literature data and ^1^H NMR spectra of both the reagent CHPTAC and the intermediary formed EPTAC (compounds A and B in Figure 1), the intense singlet at 3.24 ppm can be unambiguously assigned to the quaternary ammonium moieties introduced, more specifically, the three methyl groups attached to each of the quaternary nitrogen atoms.^23,26,35,51^ Covalent attachment to the polymeric structure of lignin is also evident from the fact that the product investigated had been subjected to exhaustive dialysis using a 2000 Da cutoff membrane, which should largely exclude low molecular compounds like CHPTAC and EPTAC from the product investigated by ^1^H NMR spectroscopy (for the full spectrum of QL-5, see Figure S2; cf. the Supporting Information). Motivated by the above-discussed discrepancy between nitrogen contents determined by elemental analysis and the presence of some signals (e.g., 3.60 and 3.61 ppm) supposedly not contributing to the ^1^H NMR spectrum by lignin, we decided to look somewhat more closely into the changes in the ^1^H NMR spectrum of pristine CHPTAC under the alkaline conditions of lignin modification and a maximum temperature of 70 °C (Figure 4). A comparison with the spectrum of pristine EPTAC confirms that the conversion of CHPTAC into EPTAC starts already at room temperature since the prominent signals of the nonsubstituted oxirane methylene group of EPTAC (ca. 3.9 and 4.0 ppm) are already clearly visible after 2 h of reaction time. Treatment of CHPTAC for the same period but at 50 °C affords full conversion of EPTAC into the ethylene glycol derivative C (Figure 1), which is known to be the major product of CHPTAC hydrolysis. However, it is evident that other products can be formed as well when the temperature is further increased to 70 °C and the time is extended to 14 h. Even though there is evidence from the ^1^H NMR spectrum that CHPTAC does not form polymeric compounds under the tested conditions, it cannot be excluded that side reactions with lignin might occur, leading to the aforementioned enhanced nitrogen contents and explaining their presence in QL-5 even after dialysis.

**Figure 5:**
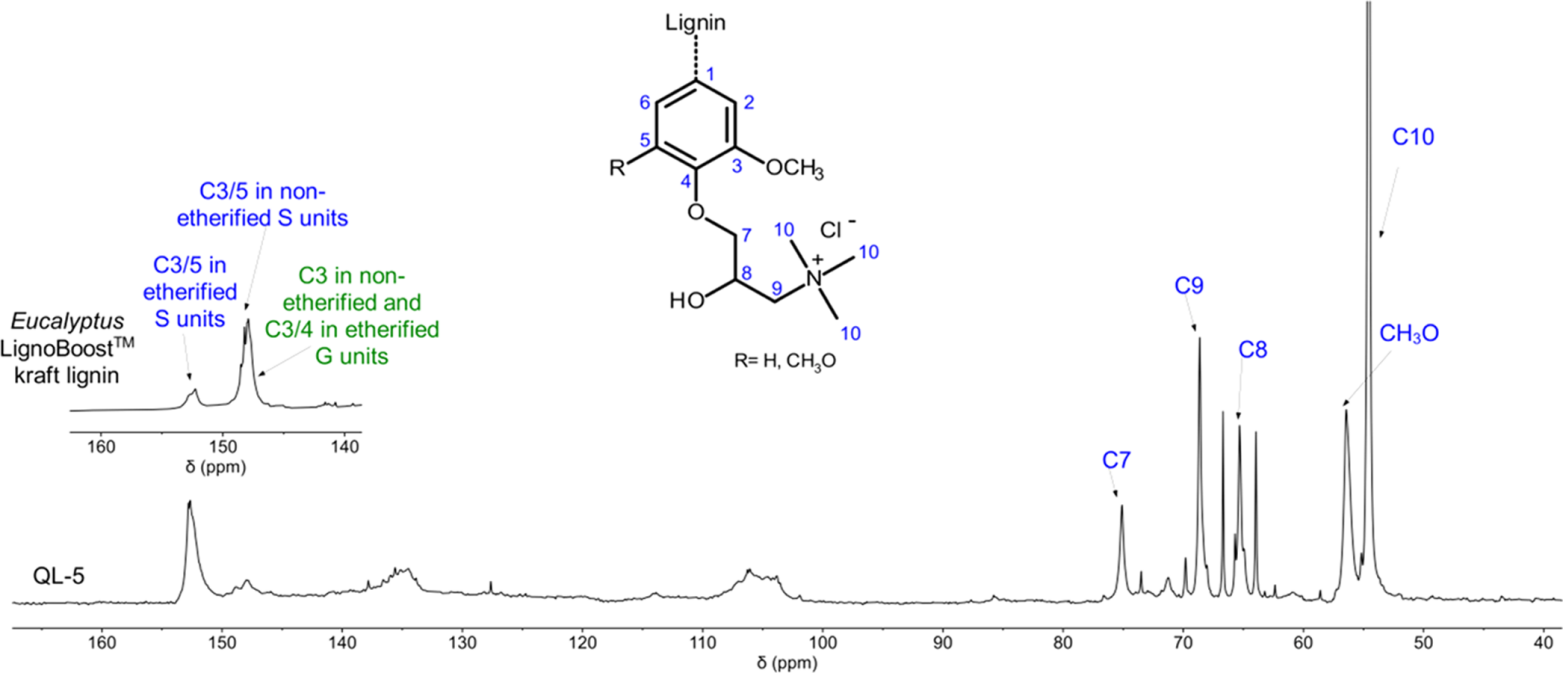
Quantitative ^13^C NMR spectrum of QL-5 with peak assignment (the inset shows the peak area between δ = 140 and 160 ppm of the parent lignin).

Quantitative ^13^C NMR spectroscopy as exemplarily conducted for sample QL-5 also confirmed the introduction of quaternary ammonium moieties (Figure 5). This can be concluded from the change of the peak pattern in the fingerprint range of 145–155 ppm where several prominent resonance signals of aromatic carbons can be found.^57^ This includes C3 atoms in nonetherified guaiacyl units (centered at 145.9 ppm^58^) as well as C3/C5 atoms in nonetherified (centered at 149.5 ppm^58^) and etherified syringyl units (centered at 152.5 ppm^59^). In view of the prevalence of syringyl units present in the parent lignin (cf. above), only changes in the peak pattern caused by conversion of S units with free phenolic groups into their etherified counterparts shall be discussed here. In the parent lignin, the integral of the peak assigned to C3/5 carbons in nonetherified S units and overlapped by C3 in G units is more than 3 times higher than that of the C3/5 atoms in etherified S moieties (150.3–153.0 ppm^47,59^).

After etherification of free phenolic groups through the reaction with the intermediary EPTAC (cf. Figure 1), this ratio is clearly reversed and the signal at 152.5 ppm is now dominant. Based on the integral ratio, a rough estimate suggests that at least 70–80% of free phenolic groups have reacted with EPTAC. Even though this value is likely to be even higher, the interference of carbon signals in nonetherified S and G units by peaks of C3 (149.4 ppm) and C4 (147.5 ppm) atoms in etherified G units does not allow for a more precise calculation. Successful modification of lignin is also concluded from the appearance of a prominent signal at 54.6 ppm, which was only found in QL-5 and was therefore—in good agreement with literature data—assigned to the methyl carbons attached to the quaternary nitrogen atoms.^35,50,51^ All carbons of the propyl spacer between the phenoxy and quaternary ammonium groups could also be assigned in good agreement with other biopolymers modified by CHPTAC or EPTAC (cf. Figure 5). The quantification of methylol groups in β-O-4 structures at 59.0–61.0 ppm^57^ did not reveal significant differences between the parent kraft lignin and QL-5. This indicates that the primary hydroxyl groups did not react with CHPTAC under the applied reaction conditions. On the other hand, a weak resonance signal at 79.5 ppm was found in the spectrum of QL-5, which was absent in the parent kraft lignin. This resonance is commonly assigned to benzylic carbon in benzyl ether structures^47^ and supports the assumption that benzylic hydroxyl groups could have been involved in lignin quaternization via a quinone methide mechanism. However, the contribution of phenolic moieties in the cationization reaction is predominant because the estimated amounts of newly formed α-O-alkyl bonds did not exceed 0.07 per aromatic ring.

**Figure 6:**
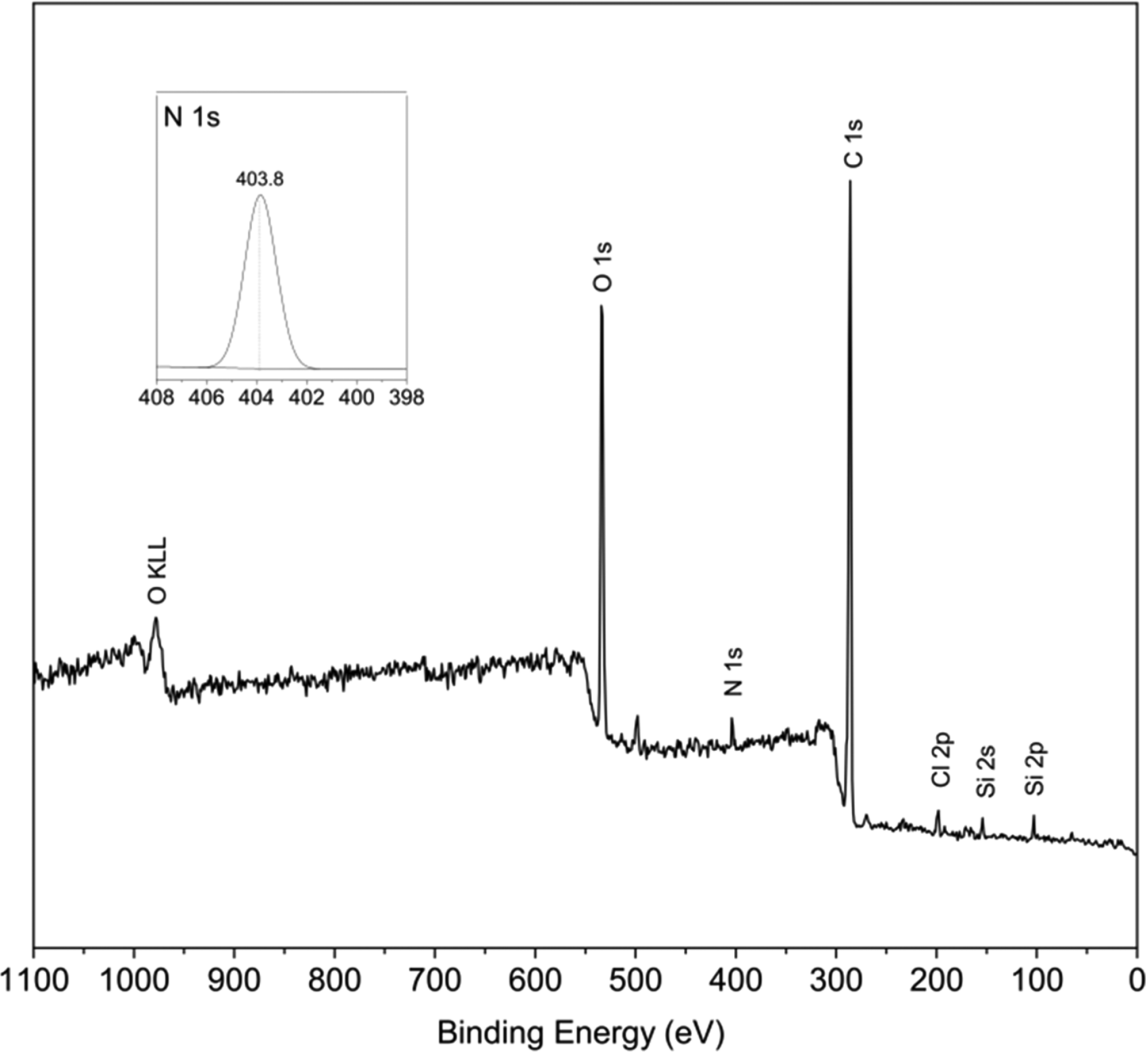
X-ray photoelectron survey spectrum of the cationic lignin QL-5.

X-ray photoelectron spectroscopy (XPS) as exemplarily conducted for QL-5 as the most interesting sample from the economic point of view (full solubility at the lowest degree of substitution) confirmed the successful introduction of quaternary ammonium moieties (Figure 6). This is evident from the binding energy of the N 1s peak centered at about 403.8 eV, which has been assigned according to the literature.^60−62^ Even though low-molecular quaternary ammonium compounds are known to have *E*_B_ values of around 402 eV, it has been shown in the example of quaternary ammonium polysulfones that cross-linking of respective linear polymers can cause the N 1s peak to shift toward higher binding energies, in that specific case almost to 403 eV.^60^ Similarly high values have been reported for pine bark chemically equipped with quaternary ammonium moieties.^61^ The relatively broad full width at half-maximum (FWHM) is probably caused by the different chemical environments of the introduced quaternary ammonium groups within the irregular lignin network structure. However, also the presence of smaller quantities of 3-(trimethylammonium)-1,2-propylenglycol oligomers formed by the ring-opening reaction of EPTAC in strongly alkaline conditions cannot be fully excluded since the N 1s binding energy would be very similar to the modified lignin (cf. discussion above about DS). It is also worth noting that cation−π interactions between remnants of unreacted CHPTAC or grafted 3-(trimethylammonium) propyl groups and the phenolic moieties of lignin could contribute to broadening of the N 1s peak. However, the likelihood of complex formation is presumably relatively low considering aspects such as high electrolyte concentration, solvent polarity, and steric hindrance of lignins, the latter being additionally promoted by the well-known π–π stacking of aromatic groups leading to expanded aggregates in the alkaline solution.^63^

There is no indication of the presence of amine moieties, which is evident from the absence of N 1s peaks in the range of 398–400 eV.^64^ Amines could have formed at elevated temperatures at the strong alkaline conditions used; however, this was not very likely to occur in an aqueous medium and at a relatively low temperature (70 °C). The overview and Cl 2p spectra also confirm the presence of an adequate quantity of chloride ions (*E*_B_ = 198.7 eV) and of some silicon, most likely introduced by alkali-aided solubilization from the used glassware.

ζ potential measurements confirmed that electrostatic repulsion of the positively charged lignin molecules and, hence, colloidal stability of respective solutions depend on the molar C/L ratio used for synthesis and, hence, on the attained degree of substitution (DS = 0.7, 1.2, 1.3, and 1.7). It was found that for the samples examined, the ζ potential increases in the order QL-2 (+12.9 ± 0.5 mV) < QL-5 (+22.4 ± 1.5 mV) < QL-7 (+24.0 ± 1.4 mV) < QL-12 (+30.3 ± 2.6 mV). This is also confirmed by surface charge calculation based on polyelectrolyte titration.^26,45^ The equivalent charge concentration (μ_eq_ L^–1^) increased in the same order QL-2 (202 ± 2) < QL-5 (494 ± 5) < QL-7 (566 ± 16) < QL-12 (697 ± 4).

**Figure 7:**
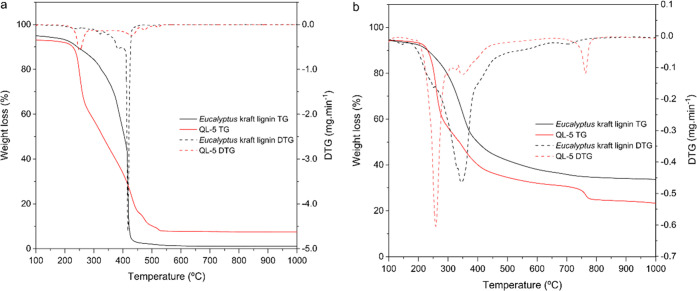
Thermostability (TG, DTG) of *E. globulus* kraft lignin and its cationic derivative (QL-5) in oxygen (a) and nitrogen atmospheres (b).

Thermogravimetric analysis conducted in both oxygen and nitrogen atmospheres for QL-5 (Figure 7) revealed a higher thermosensitivity for the cationic lignin compared to the parent material (for the DTG of cationic lignin QL-7 see Figure S3; cf. the Supporting Information). Degradation in the oxidative environment is naturally more severe than in an inert atmosphere. While under nitrogen protection, only 50 wt % QL-5 was volatilized at 400 °C, virtually quantitative gasification has occurred in the presence of oxygen. This is similar for the parent lignin and is comparable to the technical process of wood gasification.^65^ In the absence of oxygen, four prominent temperature events can be distinguished for both materials. While the release of adsorbed water until somewhat beyond 100 °C was largely similar, a significant shift of the main degradation event (corresponding to 34% mass loss) toward lower temperatures (350 °C → 260 °C) was obtained for the cationic product, which is in agreement with the literature.^66^ This is presumably due to the well-known thermal degradation of quaternary ammonium salts to afford tertiary amines and—in the case of the presence of a proton in the β-position relative to the ammonium nitrogen—an alkene by an E2 Hofmann elimination mechanism.^67^ Degradation between 300 and 380 °C accounts for about 24% weight loss. This event is presumably unaffected by quaternary ammonium moieties since the same signal was found for the nonmodified parent lignin. Considering the much higher intensity of this signal for the parent lignin, it might be concluded that free phenolic groups play a key role here. Interestingly, there is another prominent event at about 770 °C that was observed for the cationic lignin only. It is therefore assumed that heteroaromatic structures are formed here undergoing subsequent condensation and aromatization under depletion of nitrogen as is known to take place in carbonization of poly(1-acrylonitrile) fibers in the temperature range of 600–1300 °C.

### Hygroscopicity and Water Adsorption

**Figure 8:**
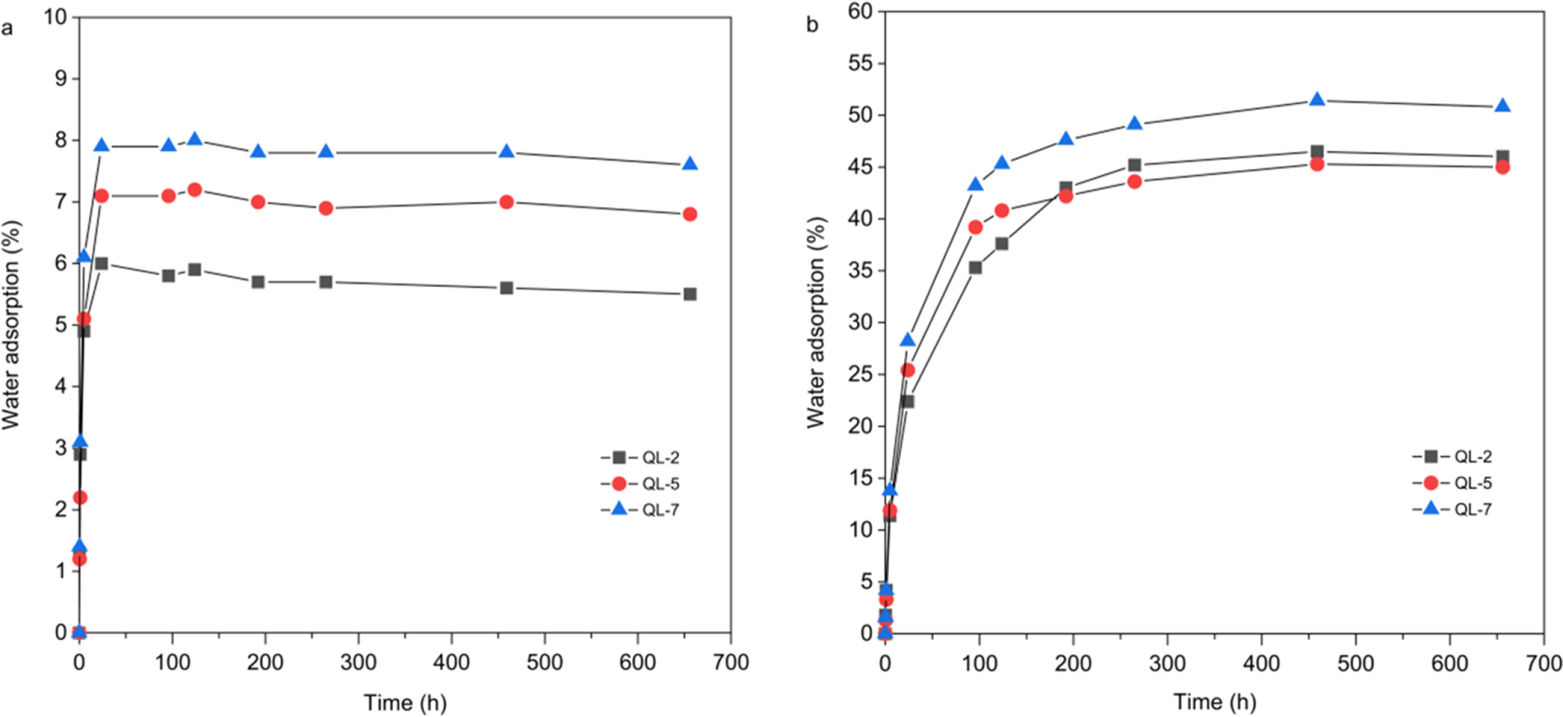
Water sorption of selected cationic hardwood LignoBoost kraft lignins (QL-2, QL-5, and QL-7) at 25 °C for 35% RH (a) and 78% RH (b).

Many quaternary ammonium salts are hygroscopic and can adsorb considerable amounts of water when stored in a humid environment. Therefore, moisture sorption was exemplarily studied for selected samples (QL-2, QL-5, QL-7), at 25 °C and two levels of relative humidity, i.e., ca. 35 and 78% RH (Figure 8). The results confirm the above assumption with regard to hygroscopicity and show that water uptake occurs quickly in both adsorption scenarios and for all materials tested. The highest adsorption capacity was found for the sample with the highest degree of substitution tested, i.e., sample QL-7 (DS 1.3). Here, the equilibrium water contents were 8 wt % (ca. 35% RH) and 50 wt % (ca. 78% RH), while the values were somewhat lower for the material of DS 0.7 (35% RH: 6 wt %; 78% RH: 45 wt %). In the example of 78% RH, it is evident that the initial adsorption is relatively fast but slows down with increasing water uptake until an equilibrium moisture content is reached after about 11 days (DS 0.7 and 1.2) and 19 days (DS 1.3). This suggests that modification and coating of material surfaces with cationic lignin not only can impart enhanced polarity to hydrophobic surfaces but might also play a beneficial role in the formation of biofilms and improved biodegradation of otherwise poorly degradable materials.

### Antioxidant Activity

**Figure 9:**
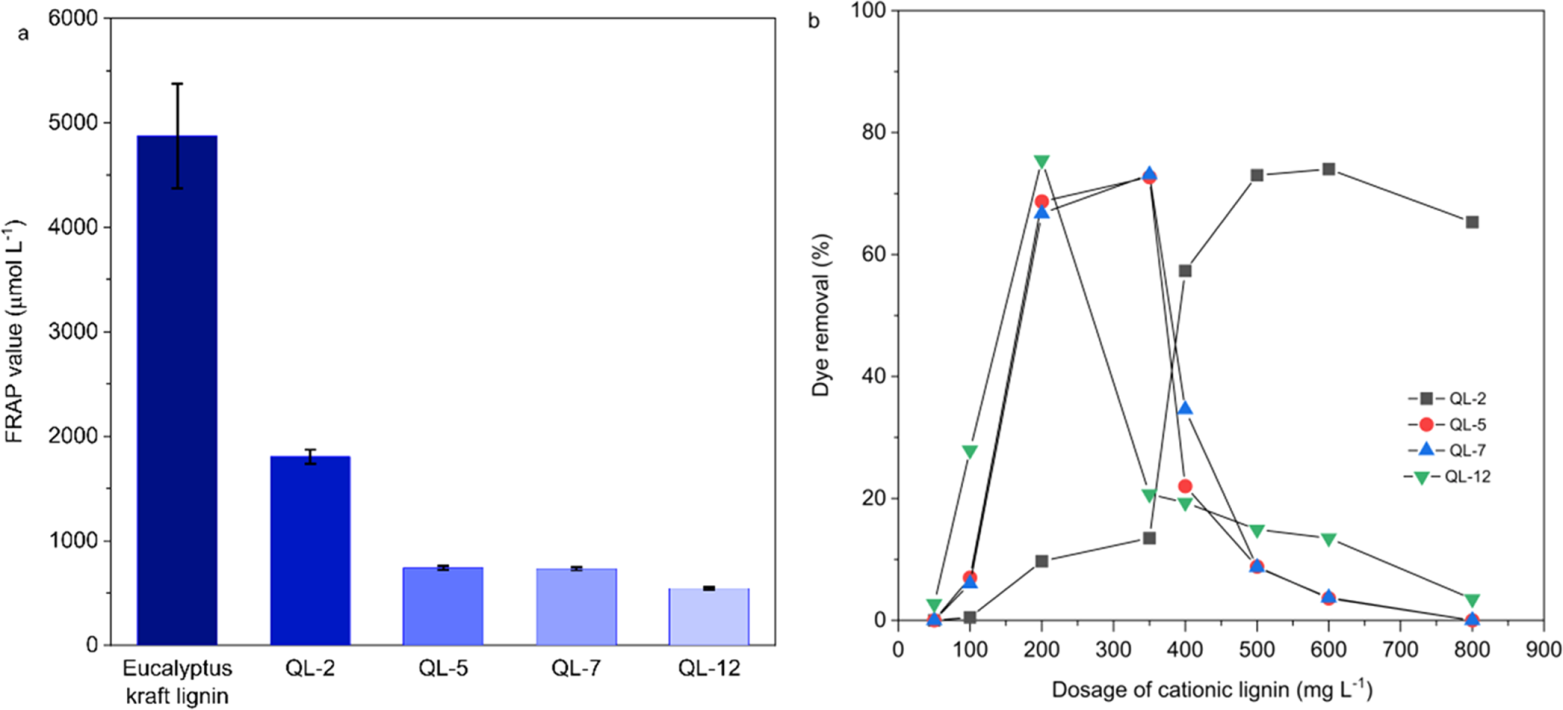
(a) FRAP antioxidant activity and (b) flocculation efficiency of selected cationic hardwood LignoBoost kraft lignins toward the anionic reactive dye Remazol Brilliant Blue R.

The successful introduction of (2-hydroxy-3-trimethylammonium)-propyl moieties has been indirectly also confirmed by the conducted ferric ion reducing antioxidant power (FRAP) antioxidant capacity assay. Polyphenols can easily transfer electrons to other substrates since they are able to delocalize, dissipate, and stabilize unpaired electrons across their conjugated π electron systems, which can eventually result in a stable oxidized state. If their “free” phenolic hydroxyl groups are once lost, such as by etherification, phenols also lose their reductive capabilities. This is confirmed by the decrease in the antioxidant activity (expressed as the FRAP value), which increased with the severity of substitution by (2-hydroxy-3-trimethylammonium)-propyl groups. According to the residual antioxidant activity of sample QL-5, about 86% of the phenolic groups have been converted into the respective ether derivative (cf. Figure 9a). This is equivalent to 0.71 phenolic groups per C9 unit and is, hence, in good agreement with the C9 phenolic group contents calculated from the SEC results (0.75). With regard to potential applications, it is worth noting that even at high degrees of substitution a residual antioxidant activity of approximately 10% exists, which could be even higher at lower degrees of substitution (Figure 9a).

### Dye Removal

The potential use of the synthesized cationic lignins as flocculation agents was tested using the anionic vinylsulfone dye Remazol Brilliant Blue R (Reactive Blue 19). The choice was based on practical considerations, as this anthraquinone derivative is used for large-scale dyeing of cotton and wool and is, hence, a major contaminant in effluents of the textile industry.^68,69^ The flocculation experiments conducted for cationic lignins of different degrees of substitution (QL-2, QL-5, QL-7, and QL-12) and using concentration series of increasing lignin-to-dye ratio (pH 7, 30 °C) confirm that the efficiency of dye removal from the solution state increases with the extent of lignin cationization. This is evident from the data compiled in Figure 9b, which show that for sample QL-12 (DS 1.7) up to 78% of the dye (*C*_Dye_ = 90 mg L^–1^) can be precipitated already at a relatively low lignin concentration (200 mg L^–1^). A similarly high value is reached for the samples QL-5 (DS 1.2) and QL-7 (DS 1.3) at a lignin concentration of 350 mg L^–1^ only. The flocculation efficiency decreases significantly at low DS and dye concentrations as suggested by the results of the sample QL-2 (DS 0.7), where a high dosage of 500 mg L^–1^ was required to precipitate about 55% of the dye from a respective solution of a lower concentration (75 mg L^–1^). It is worth noting that for each of the cationic lignins tested an optimum dosage for maximum dye removal was identified. Beyond that optimum value, the flocculation activity declines distinctly due to overcharging and restabilization of dye particles, which is in accordance with literature data.^26,32,34,42^

## Conclusions

It has been demonstrated that grafting of (2-hydroxy-3-trimethylammonium)-propyl groups onto hardwood kraft lignin is a facile approach to obtain cationic derivatives that feature excellent water-solubility (≫500 g L^–1^) independent of pH. Optimal conditions for modification of the tested *E. globulus* LignoBoost kraft lignin were as follows: CHPTAC-to-lignin molar ratio of 1.3, 3 h of reaction time in 1 M NaOH at 70 °C, and 10 wt % lignin content. The product obtained under these conditions had a DS of 1.2, a ζ potential of +22.4 mV, and a charge density of 494 μ_eq_ L^–1^. SEC, FT-IR, ^1^H NMR, ^13^C NMR, and XPS confirmed the introduction of quaternary ammonium groups. TG analyses provided evidence of a somewhat reduced thermal stability for the cationic kraft lignins at temperatures beyond 250 °C; however, this is clearly outmatched by improved compatibility with polar materials, such as biopolymer-based matrices. Furthermore, introduction of the quaternary ammonium moieties is expected to impart hardwood kraft lignin antimicrobial activity, as is known from microbicidal finishing of textiles or modification of starch using similar reagents. Respective tests are subject of ongoing investigations. FRAP antioxidant activity measurements indirectly confirmed the success of modification since increasing etherification of phenolic groups is inversely correlated with antioxidant activity. However, it is worth noting that even at high degrees of substitution, cationic lignins still exhibit some antioxidant activity. Preliminary tests suggested that cationic lignins could have potential as effective flocculants for negatively charged water pollutants, such as reactive anthraquinone dyes. Considering their high market share, conversion of kraft lignins into products featuring a pH-independent good water-solubility like lignosulfonates seems to hold great promise for promoting the utilization of this hitherto largely underutilized biopolymer in large-scale applications.

## Supporting Information Available

Additional results of FT-IR, ^1^H NMR, and DTG analyses (PDF)

## References

[ref1] Duchesne L. C.; Larson D. W. Cellulose and the evolution of plant life: The physical and biological properties of cellulose have made it the most abundant molecule in the biosphere. Bioscience 1989, 39, 238–241. 10.2307/1311160.

[ref2] Kim C.-H.; Lee J.-Y.; Park S.-H.; Moon S.-O. Global trends and prospects of black liquor as bioenergy. J. Korea TAPPI 2019, 51, 3–15. 10.7584/JKTAPPI.2019.10.51.5.3.

[ref3] Gao W.; Inwood J. P. W.; Fatehi P. Sulfonation of phenolated kraft lignin to produce water soluble products. J. Wood Chem. Technol. 2019, 39, 225–241. 10.1080/02773813.2019.1565866.

[ref4] Guo Y.; Gao W.; Fatehi P. Hydroxypropyl sulfonated kraft lignin as a coagulant for cationic dye. Ind. Crops Prod. 2018, 124, 273–283. 10.1016/j.indcrop.2018.07.078.

[ref5] Ouyang X.; Ke L.; Qiu X.; Guo Y.; Pang Y. Sulfonation of alkali lignin and its potential use in dispersant for cement. J. Dispersion Sci. Technol. 2009, 30, 1–6. 10.1080/01932690802473560.

[ref6] Yasuda S.; Hamaguchi E.; Matsushita Y.; Goto H.; Imai T. Ready chemical conversion of acid hydrolysis lignin into water-soluble lignosulfonate II: Hydroxymethylation and subsequent sulfonation of phenolized lignin model compounds. J. Wood Sci. 1998, 44, 116–124. 10.1007/BF00526256.

[ref7] Qin Y.; Yang D.; Qiu X. Hydroxypropyl sulfonated lignin as dye dispersant: Effect of average molecular weight. ACS Sustainable Chem. Eng. 2015, 3, 3239–3244. 10.1021/acssuschemeng.5b00821.

[ref8] Rivière G. N.; Korpi A.; Sipponen M. H.; Zou T.; Kostiainen M. A.; Österberg M. Agglomeration of viruses by cationic lignin particles for facilitated water purification. ACS Sustainable Chem. Eng. 2020, 8, 4167–4177. 10.1021/acssuschemeng.9b06915. 32296616 PMC7147264

[ref9] Richter A. P.; Bharti B.; Armstrong H. B.; Brown J. S.; Plemmons D.; Paunov V. N.; Stoyanov S. D.; Velev O. D. Synthesis and characterization of biodegradable lignin nanoparticles with tunable surface properties. Langmuir 2016, 32, 6468–6477. 10.1021/acs.langmuir.6b01088. 27268077

[ref10] Biswas S.; Huang X.; Badger W. R.; Nantz M. H. Nucleophilic cationization reagents. Tetrahedron Lett. 2010, 51, 1727–1729. 10.1016/j.tetlet.2010.01.094. 20204160 PMC2829780

[ref11] Bureš F. Quaternary ammonium compounds: Simple in structure, complex in application. Top. Curr. Chem. 2019, 377, 14 10.1007/s41061-019-0239-2. 31062103

[ref12] Nachtergaele W. The benefits of cationic starches for the paper industry. Starch 1989, 41, 27–31. 10.1002/star.19890410108.

[ref13] Pei A.; Butchosa N.; Berglund L. A.; Zhou Q. Surface quaternized cellulose nanofibrils with high water absorbency and adsorption capacity for anionic dyes. Soft Matter 2013, 9, 2047–2055. 10.1039/c2sm27344f.

[ref14] Littunen K.; de Castro J. S.; Samoylenko A.; Xu Q.; Quaggin S.; Vainio S.; Seppälä J. Synthesis of cationized nanofibrillated cellulose and its antimicrobial properties. Eur. Polym. J. 2016, 75, 116–124. 10.1016/j.eurpolymj.2015.12.008.

[ref15] Stepnova E. A.; Tikhonov V. E.; Babushkina T. A.; Klimova T. P.; Vorontsov E. V.; Babak V. G.; Lopatin S. A.; Yamskov I. A. New approach to the quaternization of chitosan and its amphiphilic derivatives. Eur. Polym. J. 2007, 43, 2414–2421. 10.1016/j.eurpolymj.2007.02.028.

[ref16] Pillai C. K. S.; Paul W.; Sharma C. P. Chitin and chitosan polymers: Chemistry, solubility and fiber formation. Prog. Polym. Sci. 2009, 34, 641–678. 10.1016/j.progpolymsci.2009.04.001.

[ref17] Rabea E. I.; Badawy M. E.-T.; Stevens C. V.; Smagghe G.; Steurbaut W. Chitosan as antimicrobial agent: Applications and mode of action. Biomacromolecules 2003, 4, 1457–1465. 10.1021/bm034130m. 14606868

[ref18] Peng Z.-X.; Wang L.; Du L.; Guo S.-R.; Wang X.-Q.; Tang T.-T. Adjustment of the antibacterial activity and biocompatibility of hydroxypropyltrimethyl ammonium chloride chitosan by varying the degree of substitution of quaternary ammonium. Carbohydr. Polym. 2010, 81, 275–283. 10.1016/j.carbpol.2010.02.008.

[ref19] Kotzé A. F.; Thanou M. M.; Lueßenb H. L.; de Boer A. B. G.; Verhoef J. C.; Junginger H. E. Effect of the degree of quaternization of *N*-trimethyl chitosan chloride on the permeability of intestinal epithelial cells (Caco-2). Eur. J. Pharm. Biopharm. 1999, 47, 269–274. 10.1016/S0939-6411(99)00006-5. 10382111

[ref20] Thanou M.; Florea B. I.; Geldof M.; Junginger H. E.; Borchard G. Quaternized chitosan oligomers as novel gene delivery vectors in epithelial cell lines. Biomaterials 2002, 23, 153–159. 10.1016/S0142-9612(01)00090-4. 11762833

[ref21] Laszlo J. A. Solubility and dye-binding properties of quaternized and peroxidase-polymerized kraft lignin. Environ. Technol. 1999, 20, 607–615. 10.1080/09593332008616855.

[ref22] Li Y.; Lin X.; Zhuo X.; Luo X. Poly(vinyl alcohol)/quaternized lignin composite absorbent: Synthesis, characterization and application for nitrate adsorption. J. Appl. Polym. Sci. 2013, 128, 2746–2752. 10.1002/app.38437.

[ref23] Md Noor A. M.; Mohtar S. S.; Saman N.; Busu T. N. Z. T. M.; Shaari N.; Yusoff N. A.; Mat H. Preparation of quaternized lignin derived from oil palm empty fruit bunches and its flocculation properties. J. Wood Chem. Technol. 2019, 39, 399–420. 10.1080/02773813.2019.1636823.

[ref24] Yang A.-l.; Jiang W.-j. Studies on a cationically modified quaternary ammonium salt of lignin. Chem. Res. Chin. Univ. 2007, 23, 479–482. 10.1016/S1005-9040(07)60103-2.

[ref25] Zhang Q.; Wang D.; Bei Y.; Ren S.; Fang G. Flocculation performance of trimethyl quaternary ammonium salt of lignin-sodium alginate polyampholyte. Bioresources 2013, 8, 3544–3555. 10.15376/biores.8.3.3544-3555.

[ref26] Kong F.; Parhiala K.; Wang S.; Fatehi P. Preparation of cationic softwood kraft lignin and its application in dye removal. Eur. Polym. J. 2015, 67, 335–345. 10.1016/j.eurpolymj.2015.04.004.

[ref27] Wahlström R.; Kalliola A.; Heikkinen J.; Kyllönen H.; Tamminen T. Lignin cationization with glycidyltrimethylammonium chloride aiming at water purification applications. Ind. Crops Prod. 2017, 104, 188–194. 10.1016/j.indcrop.2017.04.026.

[ref28] Wang Z.; Huang W.; Bin P.; Zhang X.; Yang G. Preparation of quaternary amine-grafted organosolv lignin biosorbent and its application in the treatment of hexavalent chromium polluted water. Int. J. Biol. Macromol. 2019, 126, 1014–1022. 10.1016/j.ijbiomac.2018.12.087. 30562516

[ref29] Hasan A.; Fatehi P. Cationic kraft lignin-acrylamide as a flocculant for clay suspensions: (1) Molecular weight effect. Sep. Purif. Technol. 2018, 207, 213–221. 10.1016/j.seppur.2018.06.047.

[ref30] Hasan A.; Fatehi P. Cationic kraft lignin-acrylamide copolymer as a flocculant for clay suspensions: (2) Charge density effect. Sep. Purif. Technol. 2019, 210, 963–972. 10.1016/j.seppur.2018.08.067.

[ref31] Wang S.; Kong F.; Gao W.; Fatehi P. Novel process for generating cationic lignin based flocculant. Ind. Eng. Chem. Res. 2018, 57, 6595–6608. 10.1021/acs.iecr.7b05381.

[ref32] Wang S.; Kong F.; Fatehi P.; Hou Q. Cationic high molecular weight lignin polymer: A flocculant for the removal of anionic azo-dyes from simulated wastewater. Molecules 2018, 23, 2005–2018. 10.3390/molecules23082005. PMC6222342 30103485

[ref33] Li R.; Gao B.; Sun S.; Yue Q.; Li M.; Yang X.; Song W.; Jia R. Amine-crosslinked lignin-based polymer: Modification, characterization, and flocculating performance in humic acid coagulation. ACS Sustainable Chem. Eng. 2015, 3, 3253–3261. 10.1021/acssuschemeng.5b00844.

[ref34] Guo K.; Gao B.; Yue Q.; Xu X.; Li R.; Shen X. Characterization and performance of a novel lignin-based flocculant for the treatment of dye wastewater. Int. Biodeterior. Biodegradation 2018, 133, 99–107. 10.1016/j.ibiod.2018.06.015.

[ref35] Li G.; Fu Y.; Shao Z.; Zhang F.; Qin M. Preparing cationic cellulose derivative in NaOH/Urea aqueous solution and its performance as filler modifier. Bioresources 2015, 10, 7782–7794. 10.15376/biores.10.4.7782-7794.

[ref36] Zheng T.; Zheng D.; Li X.; Cai C.; Lou H.; Liu W.; Qiu X. Synthesis of quaternized lignin and its clay-tolerance properties in montmorillonite-containing cement paste. ACS Sustainable Chem. Eng. 2017, 5, 7743–7750. 10.1021/acssuschemeng.7b01217.

[ref37] Liu Z.; Xu D.; Xu L.; Kong F.; Wang S.; Yang G. Preparation and characterization of softwood kraft lignin copolymers as a paper strength additive. Polymers 2018, 10, 743 10.3390/polym10070743. PMC6403858 30960668

[ref38] Vieira F. R.; Barros-Timmons A.; Evtuguin D. V.; Pinto P. C. R. Effect of different catalysts on the oxyalkylation of eucalyptus Lignoboost kraft lignin. Holzforschung 2020, 74, 567–576. 10.1515/hf-2019-0274.

[ref39] TAPPI. T211 om-02: Ash in Wood, Pulp, Paper and Paperboard: Combustion at 525 °C, 2002.

[ref40] Kavaliauskaite R.; Klimaviciute R.; Zemaitaitis A. Factors influencing production of cationic starches. Carbohydr. Polym. 2008, 73, 665–675. 10.1016/j.carbpol.2008.01.019. 26048233

[ref41] Benzie I. F. F.; Devaki M. The Ferric Reducing/Antioxidant Power (FRAP) Assay for Non-enzymatic Antioxidant Capacity: Concepts, Procedures, Limitations and Applications; John Wiley & Sons Ltd., 2018.

[ref42] Fang R.; Cheng X.; Xu X. Synthesis of lignin-base cationic flocculant and its application in removing anionic azo-dyes from simulated wastewater. Bioresour. Technol. 2010, 101, 7323–7329. 10.1016/j.biortech.2010.04.094. 20576562

[ref43] Farrell M.; Hauser P. Cationic cotton, reservations to reality. AATCC Rev. 2013, 13, 56–62.

[ref44] Wieland H.; Gattermann L.; Wieland T.; Sucrow W. Die Praxis des organischen Chemikers; De Gruyter, 2010.

[ref45] Liu Z.; Ni Y.; Fatehi P.; Saeed A. Isolation and cationization of hemicelluloses from pre-hydrolysis liquor of kraft-based dissolving pulp production process. Biomass Bioenergy 2011, 35, 1789–1796. 10.1016/j.biombioe.2011.01.008.

[ref46] Costa C. A. E.; Pinto P. C. R.; Rodrigues A. E. Evaluation of chemical processing impact on *E. globulus* wood lignin and comparison with bark lignin. Ind. Crops Prod. 2014, 61, 479–491. 10.1016/j.indcrop.2014.07.045.

[ref47] Balakshin M. Y.; Capanema E. A. Comprehensive structural analysis of biorefinery lignins with a quantitative ^13^C NMR approach. RSC Adv. 2015, 5, 87187–87199. 10.1039/C5RA16649G.

[ref48] Tejado A.; Pena C.; Labidi J.; Echeverria J. M.; Mondragon I. Physico-chemical characterization of lignins from different sources for use in phenol-formaldehyde resin synthesis. Bioresour. Technol. 2007, 98, 1655–1663. 10.1016/j.biortech.2006.05.042. 16843657

[ref49] Casas A.; Alonso M. V.; Oliet M. O.; Rojo E.; Rodríguez F. FTIR analysis of lignin regenerated from *Pinus radiata* and *Eucalyptus globulus* woods dissolved in imidazolium-based ionic liquids. J. Chem. Technol. Biotechnol. 2012, 87, 472–480. 10.1002/jctb.2724.

[ref50] Song Y.; Sun Y.; Zhang X.; Zhou J.; Zhang L. Homogeneous quaternization of cellulose in NaOH/urea aqueous solutions as gene carriers. Biomacromolecules 2008, 9, 2259–2264. 10.1021/bm800429a. 18637686

[ref51] Yan L.; Tao H.; Bangal P. R. Synthesis and flocculation behavior of cationic cellulose prepared in a NaOH/Urea aqueous solution. Clean 2009, 37, 39–44. 10.1002/clen.200800127.

[ref52] Sameni J.; Krigstin S.; Sain M. Characterization of lignins isolated from industrial residues and their beneficial uses. Bioresources 2016, 11, 8435–8456. 10.15376/biores.11.4.8435-8456.

[ref53] Pandey K. K. A study of chemical structure of soft and hardwood and wood polymers by FTIR spectroscopy. J. Appl. Polym. Sci. 1999, 71, 1969–1975. 10.1002/(SICI)1097-4628(19990321)71:12<1969::AID-APP6>3.0.CO;2-D.

[ref54] Li R.; Wang X.; Lin Q.; Yue F.; Liu C.; Wang X.; Ren J. Structural features of lignin fractionated from industrial furfural residue using alkaline cooking technology and its antioxidant performance. Front. Energy Res. 2020, 8, 83 10.3389/fenrg.2020.00083.

[ref55] Faix O. Classification of lignins from different botanical origins by FT-IR spectroscopy. Holzforschung 1991, 45, 21–27. 10.1515/hfsg.1991.45.s1.21.

[ref56] Faix O.; Beinhoff O. FTIR spectra of milled wood lignins and lignin polymer models (DHP’s) with enhanced resolution obtained by deconvolution. J. Wood Chem. Technol. 1988, 8, 505–522. 10.1080/02773818808070698.

[ref57] Evtuguin D. V.; Neto C. P.; Silva A. M. S.; Domingues P. M.; Amado F. M. L.; Robert D.; Faix O. Comprehensive study on the chemical structure of dioxane lignin from plantation *Eucalyptus globulus* wood. J. Agric. Food Chem. 2001, 49, 4252–4261. 10.1021/jf010315d. 11559119

[ref58] Timilsena Y. P.; Audu I. G.; Rakshit S. K.; Brosse N. Impact of the lignin structure of three lignocellulosic feedstocks on their organosolv delignification. Effect of carbonium ion scavengers. Biomass Bioenergy 2013, 52, 151–158. 10.1016/j.biombioe.2013.02.040.

[ref59] Li T.; Lyu G.; Liu Y.; Lou R.; Lucia L. A.; Yang G.; Chen J.; Saeed H. A. M. Deep eutectic solvents (DESs) for the isolation of Willow lignin (*Salix matsudana* cv. Zhuliu). Int. J. Mol. Sci. 2017, 18, 2266–2277. 10.3390/ijms18112266. PMC5713236 29143790

[ref60] Li G.; Pan J.; Han J.; Chen C.; Lu J.; Zhuang L. Ultrathin composite membrane of alkaline polymer electrolyte for fuel cell applications. J. Mater. Chem. A 2013, 1, 12497–12502. 10.1039/c3ta12626a.

[ref61] Zhang R.; Leiviskä T. Surface modification of pine bark with quaternary ammonium groups and its use for vanadium removal. Chem. Eng. J. 2020, 385, 123967–123977. 10.1016/j.cej.2019.123967.

[ref62] Sipponen M. H.; Smyth M.; Leskinen T.; Johansson L.-S.; Österberg M. All-lignin approach to prepare cationic colloidal lignin particles: Stabilization of durable pickering emulsions. Green Chem. 2017, 19, 5831–5840. 10.1039/C7GC02900D.

[ref63] Cheng G.; Kent M. S.; He L.; Varanasi P.; Dibble D.; Arora R.; Deng K.; Hong K.; Melnichenko Y. B.; Simmons B. A.; Singh S. Effect of ionic liquid treatment on the structures of lignins in solutions: Molecular subunits released from lignin. Langmuir 2012, 28, 11850–11857. 10.1021/la300938b. 22738225

[ref64] Mathew R.; Cooney R. P.; Malmstrom J.; Doyle C. S. AFM and angular dependent XPS studies of anchored quaternary ammonium salt biocides on quartz surfaces. Langmuir 2018, 34, 4750–4761. 10.1021/acs.langmuir.8b00535. 29597350

[ref65] Musinguzi W. B.; Okure M. A. E.; Wang L.; Sebbit A.; Løvas T. Thermal characterization of Uganda’s *Acacia hockii*, *Combretum molle*, *Eucalyptus grandis* and *Terminalia glaucescens* for gasification. Biomass Bioenergy 2012, 46, 402–408. 10.1016/j.biombioe.2012.08.001.

[ref66] Barbosa R.; Morais D. D. S.; Araújo E. M.; Mélo T. J. A. Systematic characterization of different quaternary ammonium salts to be used in organoclays. Mater. Sci. Forum 2012, 727–728, 1552–1556. 10.4028/www.scientific.net/MSF.727-728.1552.

[ref67] Zhuravlev O. E.; Nikol’skii V. M.; Voronchikhina L. I. Thermal stability of quaternary ammonium hexafluorophosphates and halides. Russ. J. Appl. Chem. 2013, 86, 824–830. 10.1134/S1070427213060062.

[ref68] Joo D. J.; Shin W. S.; Choi J.-H.; Choi S. J.; Kim M.-C.; Han M. H.; Ha T. W.; Kim Y.-H. Decolorization of reactive dyes using inorganic coagulants and synthetic polymer. Dyes Pigm. 2007, 73, 59–64. 10.1016/j.dyepig.2005.10.011.

[ref69] Mishra S.; Maiti A. The efficacy of bacterial species to decolourise reactive azo, anthroquinone and triphenylmethane dyes from wastewater: A review. Environ. Sci. Pollut. Res. 2018, 25, 8286–8314. 10.1007/s11356-018-1273-2. 29383646

