# Supplementary material for: Cationization of Eucalyptus Kraft LignoBoost Lignin: Preparation, Properties, and Potential Applications
Source: Ind Eng Chem Res. 2022 Mar 7;61(10):3503–15. doi: 10.1021/acs.iecr.1c04899 (PMC8931834; doi:10.1021/acs.iecr.1c04899)
Supplement: Supplementary file 1 — ie1c04899_si_001.pdf [file ie1c04899_si_001.pdf]

Supporting information

Additional results of FT-IR, ^1H NMR, and DTG analyses

for manuscript

Cationization of Eucalyptus kraft LignoBoost™ lignin: preparation, properties and potential applications

Pinto, P.[1,2], Magina, S.[2], Budjav, E.[3], Pinto, P.[1], Liebner, F.[2,3*], Evtuguin, D.[2]

[1] *RAIZ – Forest and Paper Research Institute, Quinta de S. Francisco, Apartado 15, 3801-501 Eixo, Aveiro, Portugal*
[2] *CICECO - Aveiro Institute of Materials and Department of Chemistry, University of Aveiro, Campus Universitário de Santiago, 3810-193 Aveiro, Portugal*
[3] *University of Natural Resources and Life Sciences, Vienna (BOKU), Department of Chemistry, Institute for Chemistry of Renewable Resources, Konrad Lorenz Straße 24, A-3430 Tulln, Austria*
** corresponding author: falk.liebner@boku.ac.at*

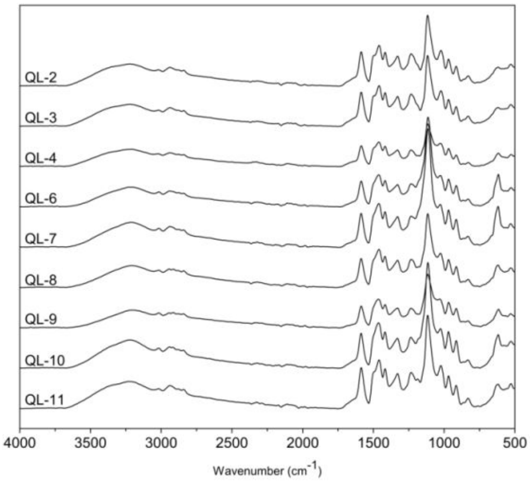

Figure S1. *FT-IR spectra of cationic lignins (QL-2 to QL-11).*

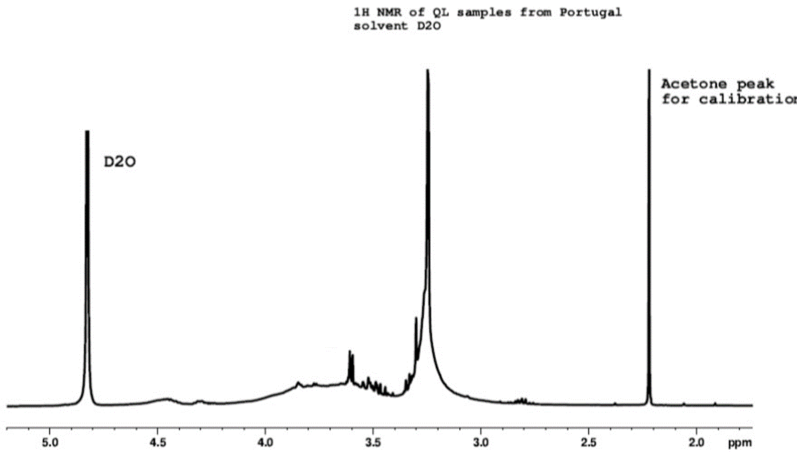

Figure S2. ^{1}H NMR full spectrum of cationized *Eucalyptus globulus* kraft lignin (QL-5).

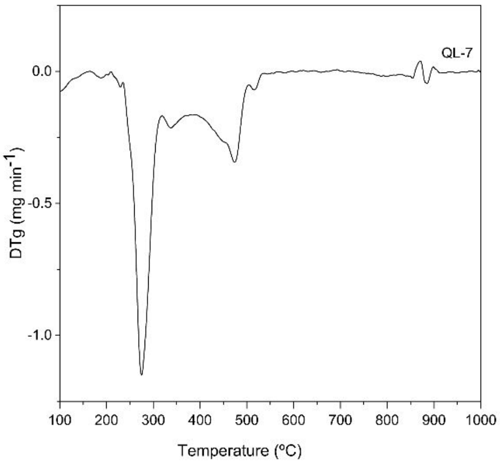

Figure S3. Thermostability (DTg) of cationic lignin (QL-7) in oxygen atmosphere.
